# Methotrexate therapy impacts on red cell distribution width and its predictive value for cardiovascular events in patients with rheumatoid arthritis

**DOI:** 10.1186/s41927-018-0012-0

**Published:** 2018-03-07

**Authors:** Julia Held, Birgit Mosheimer-Feistritzer, Johann Gruber, Erich Mur, Günter Weiss

**Affiliations:** 1 Department of Internal Medicine II, Infectious Diseases, Immunology, Rheumatology, Pneumology, Medical University of Innsbruck, Anichstr. 35, A-6020 Innsbruck, Austria; 2 Department for Physical Medicine and Rehabilitation, University of Innsbruck, Innsbruck, Austria; 3 Christian Doppler Laboratory for Iron Metabolism and Anemia Research, Innsbruck, Austria

**Keywords:** Rheumatoid arthritis, Red cell distribution width, Methotrexate, Cardiovascular events

## Abstract

**Background:**

Methotrexate (MTX) is well known to affect folic acid metabolism, so MTX treatment can result in alterations of mean corpuscular volume (MCV), which may impact on red cell distribution width (RDW), as MCV levels feed into RDW calculation. We thus questioned whether RDW levels and subsequently its diagnostic utility in RA subjects, as reported before, are influenced by ongoing MTX therapy.

We assessed the impact of disease modifying drug (DMARD) treatment, especially MTX, on RDW and evaluated their influence on the predictive value of RDW for cardiovascular (CV) events in patients with rheumatoid arthritis (RA). As far as we know, this is the first study evaluating the influence of MTX on RDW.

**Methods:**

Medical treatment, disease activity, laboratory parameters and history of CV events were retrospectively analysed in 385 RA patients at disease onset and at last follow up at our clinic. Additionally, in patients with CV event, data were recorded at last follow up prior the CV event.

**Results:**

Disease parameters and laboratory findings associated with a serious vascular event were older age (*p* < 0,001), longer disease duration (*p* = 0,002) and a higher RDW at diagnosis (*p* = 0,025). No differences in RDW levels became evident with any other treatment regimen beside MTX. MTX treated patients had significantly higher RDW compared to subjects without this drug (*p* < 0,001). In RA patients without MTX treatment, we found RDW level significantly different between those with versus without a CV event, whereas this difference disappeared in subjects receiving MTX.

**Conclusion:**

MTX impacts on RDW and might therefor reduce its prognostic value for CV events in patients taking MTX, whereas an increased RDW at diagnosis remains an early risk predictor for myocardial infarction and stroke in RA patients.

## Background

Rheumatoid arthritis (RA) is one of the most prevalent systemic inflammatory diseases which involve joints and extra-articular tissues, thereby causing organ damage. Based on the chronic inflammation and immune dysregulation, the presence of RA has been associated with cardiovascular (CV) disease and an increased CV associated mortality [1, 2]. Classical risk factors for CV disease have been investigated in RA patients, however, epidemiological studies indicate that they cannot provide a sufficient explanation for the poorer CV prognosis of RA patients as compared to non-RA subjects [3, 4]. Therefore, a combination of yet not fully elucidated factors and regulatory mechanisms may contribute to CV morbidity in RA subjects. However, it is of clinical importance to identify markers which indicate an increased CV risk or predict CV mortality in RA patients. In this regard, recent studies reported an association of elevated red cell distribution width (RDW) with CV disease in the general population [5] but also in patients with RA [6, 7].

RDW is an automated measure of the range of variation of red blood cell (RBC) volume and is calculated as the standard deviation (SD) in red blood cell size divided by the mean corpuscular volume (MCV) (RDW (%) = 1 SD of RBC volume/MCV × 100). RDW is part of the complete blood count and has traditionally been used in anemia diagnosis [8–10] and to predict the response to iron treatment [11]. A retrospective analysis of > 20.000 patients with RA indicated that higher RDW, as well as increased levels of markers of inflammation, like C-reactive protein (CRP) or erythrocyte sedimentation rate (ESR), were associated with an increased risk of a subsequent CV event in RA-patients [12].

The mechanism behind the association of elevated RDW and CV risk in RA is yet incompletely understood. Possible explanations include that RDW reflects endothelial damage and impaired vascular repair, but also that RDW mirrors vascular inflammation underlying atherosclerosis and thereby effects myocardial infarction and stroke [13]. The described positive association between IFN-alpha, a cytokine contributing to endothelial damage, and RDW would be in agreement with this concept [14].

Of note, RDW is influenced by multiple factors related to erythropoiesis, such as iron, vitamin B12 or folic acid availability, as well as by hemolysis [9]. Moreover, RDW is also affected by organ dysfunctions (e.g. liver or renal dysfunction), inflammatory activity and some specific medications [15–17]. The latter might also affect the diagnostic potential of RDW in RA patients, as these subjects are treated with numerous disease modifying antirheumatic drugs (csDMARDs), like Methotrexate (MTX), or biological DMARDs (bDMARDs), including several cytokine antibodies [18]. According to EULAR recommendations, treatment of RA should be initiated with csDMARDs, most notably MTX in combination with low dose glucocorticoids. Although low dose MTX therapy is regarded as an anchor therapy in RA, full details of its mechanism of action and off target effects are still incompletely understood [19].

Because, MTX is well known to affect folic acid metabolism, MTX treatment can result in alterations of MCV, which may impact on RDW, as MCV levels feed into RDW calculation [6]. We thus questioned, whether RDW levels and subsequently its diagnostic utility and potential in RA subjects, as reported before [7, 11, 19, 20], are influenced by ongoing MTX therapy.

## Methods

### Patients

We evaluated a total of number of 385 patients with RA. These patients were either consecutively registered in the database for evaluating iron homeostasis in RA patients (*n* = 261), or were evaluated retrospectively following their clinical examination at our outpatient clinics (*n* = 124). All patients fulfilled the 2010 ACR classification criteria for the diagnosis of RA [21]. At inclusion in the database, clinical and laboratory parameters were collected of these 261 patients. The 124 patients, who consulted the outpatients’ clinic in 2014, were retrospectively evaluated. Full blood count, disease activity parameters and medication were available from that appointment. Additionally, we retrospectively evaluated clinical and laboratory findings at last visit before a CV event occurred, this visit was defined as last follow up in these patients. In patients without a CV event, either the date the patients were included in the database or the routine follow up at our outpatient clinic was defined as last follow up. The study was approved by the Ethics committee at Medical University Innsbruck, Austria (study number AN2014-0277).

### CV-Event

The medical history was examined until Nov. 2016. A severe CV-Event was defined as myocardial infarction with or without ST-wave elevation or as an ischemic stroke. According to previous studies, CV events were clustered together [7, 22].

### RDW

RDW is mathematically calculated based on the results of a routine blood count as the one SD of RBC volume/MCV × 100 [23]. The laboratory analyses were all performed by the Central Institute of Medical and Chemical Laboratory Diagnostics, University Hospital, Innsbruck. RDW was evaluated at initial diagnosis, during follow up between 2009 and 2016 and prior to a CV event. ΔRDW showed the change between the RDW at diagnosis and prior to CV event or at last follow up in patients without CV event.

### Statistical analysis

Statistical analysis was performed using IBM SPSS Statistics 24 software. Normal distribution of laboratory parameters was assessed and retained by Kolmogorov-Smirnov-Test and One-Sample Chi-Square Test, respectively. Correlation among parameters was determined using Spearman-Rank-analysis. For comparative analysis between groups we applied Mann-Whitney-U-test, respectively cross tables. Linear regression model was applied to evaluate effects of medical treatment and laboratory findings on RDW.

## Results

We retrospectively analysed 385 RA-outpatients, of whom 77 (20%) were male and 308 (80%) female. The mean duration of RA was 14,5 years (SD 11,9), 77,2% of patients were positive for anti-citrullinated-peptide-antibodies (ACPA) and 73,5% of patients had a positive rheumatoid factor (RF) test, both parameters linked to disease severity in RA [24]. Upon evaluation of their medical history, we found that 23/385 (6%) had a documented severe CV event during the observation period (17 patients with myocardial infarction, six patients with stroke). We could not find an association between ACPA and/or RF positivity and the risk for a CV event. Disease parameters and laboratory findings associated with a vascular event were older age (*p* < 0,001) and a longer disease duration (*p* = 0,002). Patients with a cardiovascular event during follow up had a higher RDW at the date of initial diagnosis of RA as compared to subjects without a subsequent cardiovascular event (*p* = 0,025; Table 1).

**Table 1:** Demographics and laboratory parameters at initial diagnosis of RA

	CV-Event		*P*
	Yes (*n* = 23)	No (*n* = 362)	
Gender Female, n (%)	15 (65,2)	293 (80,9)	0,068
Age, mean (SD), years	74,1 (7,1)	61,8 (13,2)	< 0,001
Disease duration, mean (SD), years	23,7 (13,2)	14,1 (11,7)	0,002
RF positive, n (%)	17 (73,9)	268 (74)	0,990
ACPA positive, n (%)	18 (78,3)	279 (77,1)	0,896
RDW, mean (SD)	15,6 (0,78)	13,6 (1,28)	0,025

Demographics and laboratory parameters at initial diagnosis of RA according to a CV event during follow up, *p* values for statistical significances between the two groups are given by Mann Whitney U test, respectively cross tables, significance level at p level ≤ 0,05, *SD* standard deviation, *n* sample size, *RF* rheumatoid factor, *ACPA* anti citrullinated peptide antibodies, *RDW* red cell distribution width

We then studied for associations between disease activity, measured by DAS-28-CRP, haematological parameters (RDW and MCV at last visit or last visit before a CV event) and csDMARDs therapy in patients with or without a CV event. While no significant difference was seen for laboratory parameters, disease activity and all other medications including biological DMARDs between patients with and without a subsequent CV event, we found that leflunomid was administrated more frequently in patients with CV events, however, the total number of patients receiving leflunomid was very low (*p* = 0,05; Table 2) .

**Table 2:** Clinical and laboratory findings during follow up

	CV-Event		*P*
	Yes (*n* = 23)	No (*n* = 362)	
DAS 28 at follow up, mean (SD)	3,12 (0,93)	2,82 (1,37)	0,252
MCV at follow up, mean (SD)	86,6 (5,97)	87,2 (5,7)	0,533
RDW at follow up, mean (SD)	15,1 (2,2)	14,3 (1,5)	0,130
DMARDs at follow up, n (%)			
Methotrexate	15 (65,2%)	207 (57,2%)	0,451
Sulfasalazine	0	9 (2,5%)	0,445
Hydroxychloroquine	2 (8,7%)	27 (7,5%)	0,868
Leflunomide	3 (13%)	15 (4,1%)	0,050
Azathioprine	0	6 (1,7%)	0,533
Glucocorticoids	8 (34,8%)	133 (37,5%)	0,810
bDMARD	8 (34,8%)	130 (36%)	0,892

Clinical and laboratory findings during follow up associated with CV event, significance level at p level ≤ 0,05 –see legend to Table 1, *DAS 28* disease activity score 28, *MCV* mean corpuscular volume, *(b)DMARD* (biological) disease modifying antirheumatic drug

Out of 385 RA patient included in the evaluation 284/385 (73,8%) were under treatment with csDMARDs and 83/284 (29,2%) patients were under combination therapy with biological DMARDs and csDMARDs. Because certain csDMARDs may impact on erythropoiesis or modulate the availability of factors involved in erythropoiesis, such as folic acid [25], we next studied for differences in RDW according to underlying therapy. Whereas no differences in RDW levels became evident with any other DMARD treatment, patients receiving MTX therapy had significantly higher RDW as compared to subjects without this drug (*p* < 0,001), although all patients under MTX treatment had folic acid supplementation prescribed (Table 3).

**Table 3:** RDW distribution as a function of underlying DMARD therapy

DMARD	Intake	*n*	RDW, mean (SD)	*p*
Methotrexate	Yes	222	14,5 (1,44)	< 0,001
	No	163	14,0 (1,55)	
Sulfasalazine	Yes	9	13,7 (1,56)	0,119
	No	376	14,3 (1,5)	
Hydroxychloroquine	Yes	30	13,9 (1,19)	0,085
	No	355	14,4 (1,52)	
Leflunomide	Yes	18	14,7 (2,06)	0,429
	No	367	14,3 (1,47)	
Azathioprine	Yes	6	14,7 (1,23)	0,273
	No	379	14,3 (1,51)	
bDMARDs	Yes	139	14,2 (1,45)	0,381
	No	246	14,4 (1,53)	

RDW distribution as a function of underlying DMARD therapy, significance level at p level ≤ 0,05, *bDMARDs* biological DMARDs, see tables above, *n* number of patients in respective groups

**Fig. 1:**
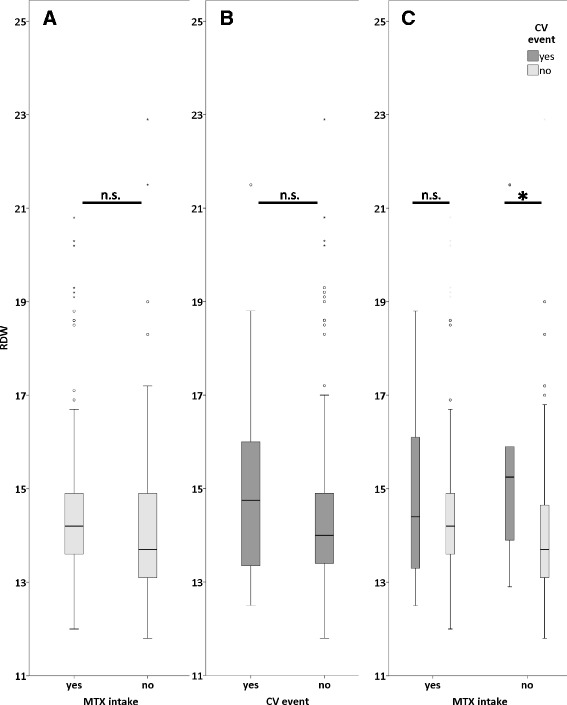
Differences in RDW levels in patients with CV events and with/without MTX; **a** RDW at last follow up in all RA patients notwithstanding concomitant treatment, depending on CV-events (yes: *n* = 23, no: *n* = 362). **b** RDW in all patients independent of CV events, depending on MTX-treatment (yes: *n* = 222, no: *n* = 163). **c** RDW in patients with and without MTX intake conditional to CV events. (MTX yes+CV yes: *n* = 15, MTX yes+CV no: *n* = 207, MTX no+CV yes: *n* = 8, MTX no+CV no: *n* = 155), n.s. not significant at *p*-level 0,05, *: *p* = 0,006 as determined by Mann Whitney U test

We then studied whether or not MTX treatment had an effect on the predictive value of RDW for severe CV events. The last routine follow up of patients with evaluation of laboratory parameters prior to the CV events was 82 days (mean) prior to such a CV complication. No significant differences in RDW, hemoglobin levels, CRP or ΔRDW (change in RDW between initial diagnosis and follow up prior to the CV event/last patient visit) were found in our cohort between patients incurring a CV event or not. Of note, we found a highly significant difference in RDW levels at last follow up, see definition above, in subjects without MTX treatment which was absent in subjects under MTX treatment comparing patients with and without a CV event (Fig. 1). Moreover, MTX-treated patients had significantly higher RDW levels than patients without MTX therapy (Table 4).

**Table 4:** Effects of MTX intake on laboratory parameters at last follow up

	MTX-use		*p*	CV-Event		*p*
	Yes (*n* = 222)	No (*n* = 163)		Yes (*n* = 23)	No (*n* = 362)	
RDW, mean(SD), %	14,5 (1,4)	14,1 (1,6)	< 0,001	15,05 (2,2)	14,25 (1,4)	0,116
Hb, mean(SD), g/l	134,8 (14,5)	132,9 (14)	0,101	130,2 (14,4)	134,2 (14,3)	0,058
CRP, mean(SD), mg/dl	0,65 (0,97)	1,03 (2,1)	0,559	0,99 (1,1)	0,8 (1,6)	0,060
ΔRDW, mean(SD)	0,53 (1,24)	0,23 (1,0)	0,057	0,45 (0,6)	0,43 (1,2)	0,809

Effects of MTX intake on laboratory parameters at last visit prior to the CV event/last follow up. *ΔRDW* Change in RDW between initial diagnosis and follow up prior to the CV event/last follow up in patients without CV event. *Hb* hemoglobin level. In the overall cohort RDW at established disease was not applicable as predictive marker for a CV event. A tendency with lower hemoglobin levels and higher CRP was shown. MTX intake significantly affected RDW

In patients without MTX therapy RDW was significantly higher in those with a subsequent CV event. (*p* = 0,006; Fig. 1). This predictive value of RDW was abolished in patients taking MTX (*p* = 0,448, Fig. 1).

Multiple linear regression analysis confirmed the relationship between RDW prior to the CV event and MTX treatment as well as associations of haemoglobin levels and age with RDW (Table 5A).

**Table 5:** A) multiple linear regression modelling the relationship with RDW. B) Binary regression relationship between CV events and RDW

A: Multiple linear regression			B: Binary regression			
Dependent variable: RDW at follow up			CV-Event			
	*p*	95% CI	MTX		*p*	95%CI
MTX	< 0,001	0,290–0,818	no	RDW follow up	0,018	0,491–0,935
Age, y follow up	< 0,001	0,011–0,031	yes	RDW follow up	0,511	0,624–1,247
Hb, g/l follow up	< 0,001	−0,057–−0,039				

A) Multiple linear regression modelling the relationship with RDW at follow up, B) Binary regression, relationship between CV events and RDW at follow up according to MTX intake, *y* years, *Hb* hemoglobine level, *MTX* methotrexate, *CV* cardiovascular, *CI* confidence interval

When performing binary regression for the risk of a CV event, we found that MTX naïve patients had a significant correlation between RDW and a CV event (Table 5B) which was not true for patients receiving MTX.

## Discussion

As far as we know, this is the first study evaluating the influence of csDMARDs on RDW. MTX impacts on RDW hence on the predictive value of RDW for CV events. Previous studies suggest that RDW is a good prognostic marker for CV disease and survival, but none of them evaluated concomitant treatment [6, 7, 12, 17]. This was also confirmed in our study indicating that an enhanced RDW at initial diagnosis, but neither ACPA nor RF positivity, is associated with an increased risk of a severe CV event [26].

However, we found that the predictive potential of RDW during follow up largely depends on the treatment of patients. Specifically, we identified that the diagnostic value of RDW as a risk indicator for subsequent CV disease is abolished in RA patients receiving MTX therapy. Although MTX is regarded as the anchor drug in RA, the mechanism of action is incompletely understood and it has been associated with negative effects on hematopoiesis, mainly via its impact on folic acid pathways, but also via direct toxic effects on hematopoietic progenitors. Accordingly, MTX but neither other csDMARDs nor bDMARD treatment resulted in alterations of red blood cell volume (MCV) and haemoglobin content of erythrocytes [27, 28]. To avoid such negative effects, patients under MTX therapy are supplemented with folic acid which was also the case in our subjects under MTX treatment [29]. However, RA patients under MTX treatment had increased RDW and MCV levels as compared to RA patients without MTX. It remains to be clarified, whether this can be referred to effects of MTX not linked to folic acid deficiency, or a reduced compliance of patients in regard to folic acid supplementation, which we could not study, because incomplete results of folic acid determination in blood were available in our study cohort. However, while MTX treatment resulted in a loss of the predictive value of RDW for subsequent CV events, higher RDW at initial diagnosis of all patients, at follow up prior to a CV event in patients without MTX treatment were significantly associated with an increased risk for a CV event.

Our study has limitations because of the retrospective design, the low number of patients with CV events and the fact that we had only insufficient data to evaluate the association of RDW, MTX therapy and CV events with other important variables including classical CV factors, the implication of a genetic component, or iron homeostasis [11, 30, 31]. In this regard, patients with RA who carried the methylene tetrahydrofolate reductase (MTHFR) 1298 allele C frequency were previously found to have an increased frequency of CV events after 5 and 10 years of follow-up. Moreover, patients carrying the MTHFR 1298 AC and CC genotypes had a significantly decreased flow-mediated endothelium-dependent vasodilatation, a marker of endothelial dysfunction that is an early indicator of atherogenesis, when compared with those carrying the MTHFR 1298 AA genotype [32]. More recent results also indicate that MTHFR expression is significantly reduced in patients with RA compared to controls. It was found to be especially true for RA patients with ischemic heart disease [33]. Taken these considerations together, these results indicate that MTHFR gene may influence the risk of subclinical atherosclerosis and CV disease in patients with RA.

Therefore, we need prospective evaluations of such risk markers and profiles to gain further insights into the functional and diagnostic role of RDW and alterations of hematological parameters as risk predicators for CV events in patients with RA. Accordingly, such confirmation may also translate into clinical practice, because patients at a higher risk, based on increased RDW may deserve an intensified clinical follow up with an improved control of classical CV risk factors, such as lipid status, hypertension, smoking status or hyperuricemia.

## Conclusion

Our study approves RDW at initial diagnosis of RA as a risk predictor for serious CV events but also indicates that its predictive value is lost during follow up in patients receiving MTX therapy, whereas it remains valid in subjects receiving non-MTX containing treatments.

## Abbreviations

ACR: American College of Rheumatology
CRP: C-reactive protein
CV: Cardiovascular
DAS-28: Disease activity score 28
DMARD: Disease modifying drugs
ESR: Erythrocyte sedimentation rate
EULAR: European League against Rheumatism
MCV: Mean corpuscular volume
MTHFR: Methylene tetrahydrofolate reductase
MTX: Methotrexate
RA: Rheumatoid arthritis
RDW: Red cell distribution width
SD: Standard deviation
